# Optical Coherence Tomography Reflectivity in Foveal Cysts: A Novel Biomarker for Early-Response Prediction of Diabetic Macular Edema Treated with Dexamethasone

**DOI:** 10.3390/life12101475

**Published:** 2022-09-23

**Authors:** Daniel Duck-Jin Hwang

**Affiliations:** 1 Department of Ophthalmology, Hangil Eye Hospital, Incheon 21388, Korea; daniel.dj.hwang@gmail.com; Tel.: +82-32-503-3322; 2 Department of Ophthalmology, Catholic Kwandong University College of Medicine, Incheon 22711, Korea

**Keywords:** central macular thickness, dexamethasone, diabetic macular edema, spectral-domain optical coherence tomography

## Abstract

This study investigated spectral-domain optical coherence tomography (OCT) biomarkers to predict short-term anatomical improvement for diabetic macular edema (DME) after dexamethasone (DEX) injection in intravitreal anti-vascular endothelial growth factor (anti-VEGF) non-responders. This retrospective comparative study included 31 eyes of 31 patients with DME unresponsive to anti-VEGF, divided into better and lesser responder groups. OCT prior to DEX injection was used to evaluate the morphological features including optical density (ODN) and optical density ratio (ODR) of the outer nuclear layer (ONL) cysts. Correlations between baseline OCT parameters and mean central macular thickness (CMT) changes at 1 month were analyzed. There were no between-group differences in age, sex, number of previous anti-VEGF injections, duration of diabetes, or HbA1c level. However, ODN and ODR values in ONL cysts were lower in the better responder group (*p* = 0.020 and *p* < 0.001, respectively). ODN and ODR showed negative correlations with CMT changes (*R* = −0.546, *p* = 0.002 and *R* = −0.436, *p* = 0.014, respectively). Higher OCT reflectivity in the foveal cystoid space was associated with a lesser decrease in CMT after DEX injection in anti-VEGF non-responders, suggesting the usefulness of this parameter in predicting short-term CMT responses after DEX injection.

## 1. Introduction

Intravitreal anti-vascular endothelial growth factor (anti-VEGF) injection has been the first-line therapy for diabetic macular edema (DME), and it has been reported to be effective in improving visual acuity (VA) and reducing central retinal thickness [1]. However, approximately 40% of patients with DME show no or poor response to anti-VEGF treatment [2,3]. In these cases, it is widely known that intraocular steroid injection for DME effectively improves the anatomy and VA. Furthermore, several studies have reported the effectiveness of steroids in DME unresponsive to anti-VEGF therapy [4,5,6,7,8,9].

In studies reporting the effectiveness of intraocular steroid injection in DME refractory to anti-VEGF therapy, results have often been quantified using optical coherence tomography (OCT). Following intraocular triamcinolone injection (IVTA), disorganization of the retinal inner layers (DRIL), intraretinal cyst location and size, hyperreflective foci (HRF), external limiting membrane (ELM) integrity, ellipsoid zone (EZ) integrity, and the presence of subretinal fluid (SRF) on OCT observed prior to IVTA have shown associations with functional or anatomical improvement [10,11,12]. In contrast, studies that investigated the effect of intravitreal dexamethasone (DEX) injection reported that DRIL, cyst location and size, HRF, ELM integrity, EZ integrity, and SRF showed functional improvements in various elements including VA [13,14,15,16]. However, few studies on OCT biomarkers associated with anatomical improvement have been reported [17,18]. Furthermore, to the best of my knowledge, few studies have reported OCT biomarkers in relation to anatomical improvement in anti-VEGF non-responders by dividing them into responders and non-responders after DEX injection [19,20,21,22].

The presence of an OCT biomarker that can predict the anatomical response of an anti-VEGF non-responder to DEX injection will provide an important clue when deciding whether to switch from anti-VEGF to DEX treatment in clinical practice. Therefore, this study focused on identifying an OCT biomarker that can predict short-term anatomical improvement after DEX injection in anti-VEGF non-responders.

## 2. Materials and Methods

### 2.1. Study Design and Ethics

In this retrospective, interventional, comparative study, the electronic medical records of patients diagnosed with DME between July 2018 and January 2019 at Hangil Eye Hospital were reviewed. This study was conducted according to the guidelines of the Declaration of Helsinki of 1975 and approved by the Institutional Review Board (IRB) of Hangil Eye Hospital. The requirement to obtain informed consent from study participants was waived by the Hangil Eye Hospital IRB given the retrospective nature of the study.

### 2.2. Participants

Patients were included if they: (1) had DME involving the fovea, (2) had a central macular thickness (CMT) > 300 μm, and (3) did not respond to intravitreal bevacizumab injections (IVBs). DME was defined as refractory to IVB if CMT did not decrease by >50 μm and remained >300 μm after three consecutive monthly IVB injections. Exclusion was based on the following criteria: (1) previous intravitreal DEX, posterior subtenon TA injection, or IVTA; (2) a history of uveitis or glaucoma; (3) retinal arterial occlusion or retinal vein occlusion; (4) epiretinal membrane or vitreomacular traction; (5) any retinal disease other than diabetic retinopathy; (6) previous focal or grid laser treatment; (7) panretinal photocoagulation treatment <6 months before the first DEX injection; (8) a history of pars plana vitrectomy; and (9) any other intraocular surgery within the last 6 months.

One month after DEX injection, patients were classified into the better responder and lesser responder groups. Patients were allocated to the better responder group if their CMT value decreased to <300 μm or if the reduction was >200 μm following DEX injection. Patients who did not meet these criteria were allocated to the lesser responder group.

### 2.3. Ophthalmic Examinations

Ophthalmologic examinations including slit-lamp examination, fundoscopy, best-corrected visual acuity (BCVA), intraocular pressure (IOP), and CMT were measured at the initial visit (baseline) and 1 month after DEX injection. The average thickness of all points within the inner 1 mm circle was defined as the CMT of the fovea, based on the subfields used in the Early Treatment Diabetic Retinopathy Study. CMT was measured using spectral-domain OCT (SD-OCT) (Spectralis OCT, Heidelberg Engineering, Heidelberg, Germany). OCT images of patients were obtained for all 25 horizontal section lines that were 250 μm apart from each other.

SD-OCT images obtained at baseline and 1 month after DEX injection were qualitatively and quantitatively assessed for the presence of several morphologic features, including: (1) DRIL within the foveal 1000 μm [18,23]; (2) cystic changes in the inner nuclear layer (INL); (3) cystic changes and maximal cyst size (mild < 100 μm, 100 ≤ moderate < 200 μm, severe ≥ 200 μm) in the outer nuclear layer (ONL); (4) ELM integrity (normal, partly disrupted, moderately disrupted, completely disrupted); (5) EZ integrity (normal, partly disrupted, moderately disrupted, completely disrupted); (6) presence and quantity of HRF (absent, few < 10, many ≥ 10); and (7) presence of SRF. These features were evaluated on five horizontal OCT scans: one B-scan encompassing the fovea and four B-scans (250 and 500 mm superior to, and 250 and 500 mm inferior to the fovea).

**Figure 1:**
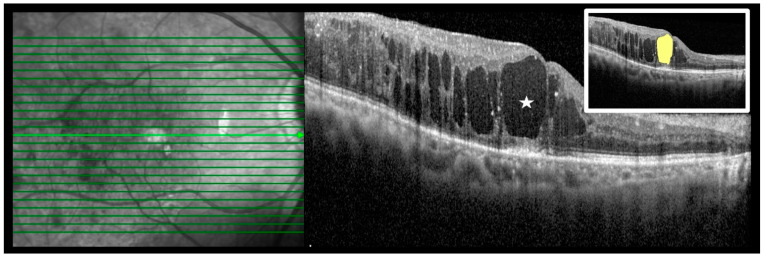
Measurement of optical coherence tomography reflectivity expressed as the optical density (ODN) of the cystoid space in the outer nuclear layer (ONL) on optical coherence tomography images obtained using ImageJ software and calculation of the optical density ratio (ODR) of the ONL cyst. After selecting the entire cyst region, the mean ODN was calculated; the entire vitreous area was selected and the average pixel intensity within the vitreous cavity was calculated in the same manner. To obtain the ODR, the average pixel intensity of the cyst was divided by that of the vitreous cavity. ODN of the ONL cyst (asterisk, yellow area in the white box) = 43.96, ODR = 3.74. Central macular thickness decreased from 707 μm to 364 μm 1 month after the intravitreal dexamethasone injection.

Reflectivity of the cystic spaces in the ONL was also measured, as previously described [24,25]. The largest cystic space within 1000 μm of the fovea was manually circumscribed for each patient, and the average reflectivity in this area was measured using ImageJ software (http://imagej.nih.gov/ij/; provided in the public domain by the National Institutes of Health, Bethesda, MD, USA, accessed 22 September 2021) [25]. The entire cystic space in the ONL and vitreous area on the same line were selected to measure the optical density (ODN). The ODNs were extracted from the measured gray-level intensity of the corresponding region on a scale from 0 (pure black) to 255 (pure white). The selected areas were measured in pixels. Subsequently, the author calculated the relative reflectivity scores (optical density ratio, ODR = ODN of the ONL cyst/ODN of the vitreous) using the reflectivity levels of the vitreous cavity as a standard in each image (Figure 1).

### 2.4. Statistical Analysis

Statistical analyses were performed using a commercially available software package (SPSS Statistics version 23; IBM Corp., Armonk, NY, USA). The Mann–Whitney U test and Fisher’s exact test were performed to compare the characteristics of the two patient groups. Pearson correlation analysis was performed to evaluate the relationship between ODN/ODR and CMT changes. Paired *t*-tests were used to compare CMT and BCVA before and after DEX injection. A two-sided *p*-value was used, and statistical significance was set at *p* < 0.05.

## 3. Results

**Figure 2:**
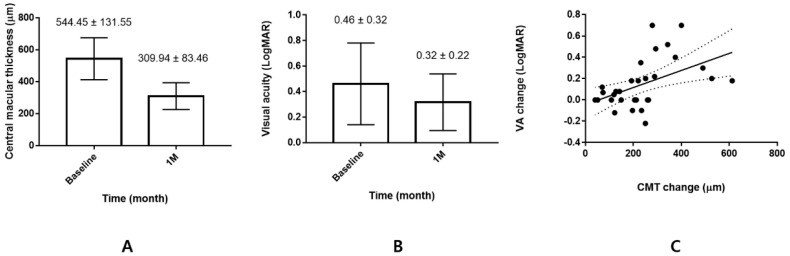
Changes of central macular thickness and visual acuity. (**A**) Central macular thickness (CMT) decreased from 544.45 ± 131.55 μm to 309.94 ± 83.46 μm 1 month after intravitreal dexamethasone injection (*p* < 0.001). (**B**) Visual acuity (VA) improved from 0.46 ± 0.32 logMAR to 0.32 ± 0.22 logMAR after injection (*p* = 0.001). (**C**) Visual gain after injection was associated with CMT change (Pearson’s correlation coefficient *R* = 0.485, *p* = 0.006).

A total of 31 eyes of 31 patients with DME were included in this study. The baseline characteristics are presented in Table 1. The mean age was 58.77 ± 10.32 years; duration of diabetes, 9.94 ± 9.00 years; HbA1c, 7.47 ± 1.31%; and number of previous anti-VEGF injections, 4.95 ± 2.37. Further, the mean baseline BCVA of 0.46 ± 0.32 logMAR improved to 0.32 ± 0.22 logMAR 1 month after DEX injection (*p* = 0.003, Figure 2). The mean baseline IOP of 15.42 ± 2.69 mmHg increased to 17.48 ± 5.25 mmHg 1 month after DEX injection (*p* = 0.040).

### 3.1. Comparison of Clinical Characteristics between the Better and Lesser Responder Groups

The mean CMT reduction after DEX injection was 234.52 ± 137.68 μm; all eyes except for one (40 μm decrease) showed a CMT reduction of ≧50 μm (Figure 2). Of the 31 participants, 22 (71%) and 9 (29%) were categorized into the better and lesser responder groups, respectively. There were no between-group differences in the clinical characteristics (Table 2).

### 3.2. Comparison of OCT Parameters between the Better and Lesser Responder Groups

Baseline CMT was higher in the better responder group (577.68 ± 138.95 μm vs. 463.22 ± 61.27 μm, *p* = 0.020); one month after DEX injection, CMT decreased more in the better responder group than in the lesser responder group (290.36 ± 123.31 vs. 98.00 ± 40.37, respectively; *p* < 0.001). There were no significant between-group differences in baseline DRIL, presence of cysts in the INL and ONL, ELM and EZ integrity, presence of SRF, or HRF number. However, there were significant between-group differences in ODN (*p* = 0.020) and ODR (*p* < 0.001) in ONL cysts, with values lower in the better-responder group (Table 3).

### 3.3. Correlation between Optical Density in ONL Cysts or Optical Density Ratio and CMT or VA Change

The mean baseline ODN in ONL cysts was 44.83 ± 19.76, and ODN values were lower in the better responder group than those in the lesser responder group (38.54 ± 15.24 vs. 60.22 ± 21.88, respectively; *p* = 0.020). Further, the mean baseline ODR was 2.10 ± 1.33 which was lower in the better responder group than that in the lesser responder group (1.55 ± 0.88 vs. 3.46 ± 1.26 μm, respectively; *p* < 0.001).

**Figure 3:**
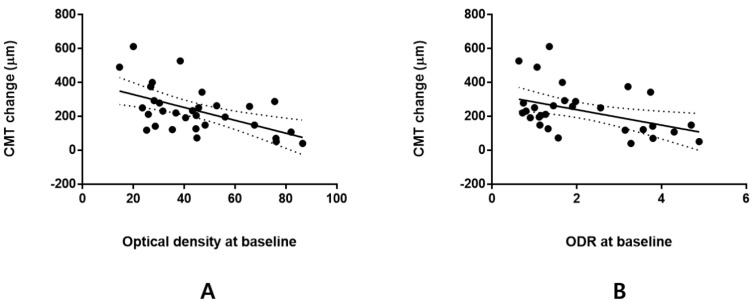
Correlation between optical density (**A**) or optical density ratio (**B**) and central macular thickness. Optical density at baseline was significantly associated with central macular thickness (CMT) change 1 month after intravitreal dexamethasone (DEX) injection (Pearson’s correlation coefficient *R* = −0.546, *p* = 0.002). The optical density ratio at baseline was also significantly correlated with CMT change 1 month after DEX injection (*R* = −0.436, *p* = 0.014).

In cases of higher baseline ODN and ODR, a lesser CMT decrease was observed 1 month after DEX injection. Further, the better responder group had a lower baseline ODN and ODR; hence, ODN and ODR baseline values showed a significant negative association with CMT change 1 month after DEX injection (Pearson correlation coefficient *R* = −0.546, *p* = 0.002 and *R* = −0.436, *p* = 0.014, respectively; Figure 3). However, the baseline ODN and ODR values were not associated with VA changes 1 month after DEX injection (*p* = 0.070 and *p* = 0.274, respectively).

## 4. Discussion

This study aimed to identify OCT biomarkers that can predict short-term anatomical improvement after DEX injection in anti-VEGF non-responders with DME. When patients were divided into two groups based on CMT change 1 month after DEX injection, the following baseline OCT parameters were not found to be related to the degree of CMT improvement: the degree of DRIL, INL and ONL cysts, and ELM and EZ integrity. However, the OCT reflectivity of ONL cysts, expressed as ODN or ODR, was significantly negatively associated with CMT changes. In other words, higher OCT reflectivity was associated with lesser improvement in CMT.

Since larger baseline OCT reflectivity of ONL cysts was associated with a smaller decrease in CMT after 1 month, the association between higher OCT reflectivity and less improvement in VA was expected. However, baseline ODN and ODR values and the degree of VA improvement after 1 month were not related. The degree of VA improvement is affected by CMT reduction and various factors related to visual signal transduction (ELM and EZ integrity, inner retinal integrity without DRIL); this may explain why ODN and VA changes were unrelated [26,27,28].

This study showed that OCT baseline reflectivity of ONL cysts could predict DME improvement 1 month after DEX injection; however, further studies are needed to investigate the relationship between OCT reflectivity of foveal cysts and VA change, CMT change pattern, and recurrence over a long-term follow-up period. In a previous study that observed the effects of IVTA or posterior subtenon TA injection in DME, rebounding macular thickening was reported to occur less in the group with a high baseline reflectivity in the foveal cystoid space, and VA deterioration was also less for 6 months after injection [29]. In other words, lower OCT reflectivity in the foveal cystoid spaces was associated with rebounding macular thickening and VA deterioration. Although this was not the main result of their study, in Figure 4B presented in their report, when OCT reflectivity in the foveal cystoid space was divided into four groups according to the value, the group with the highest reflectivity score exceeding 30 had the least DME reduction in 1 month after TA injection. This result is similar to that of the present study, in which a higher OCT reflectivity showed lesser improvement in CMT. However, the study did not target anti-VEGF non-responders, and the drugs used were also different from those in this study (TA vs. DEX); thus, the interpretation of the results requires attention. In addition, the method of measuring OCT reflectivity in the cystoid space also differed from that used in the present study [29,30].

The exact mechanisms controlling OCT reflectivity in the cystic space are not yet known. It has been reported that there is an association between OCT reflectivity of foveal cysts and the breakdown of the blood–retinal barrier [30]. Horri et al. have also shown that small molecules, including water, seem to leak into the cystic spaces through an intercellular junctional complex, depending on differences in hydrostatic pressure [29]. In cases of lower OCT reflectivity, they assumed that the differences in hydrostatic pressure between the cystic spaces and retinal capillaries would induce extravasation of smaller molecules into the cystic spaces, leading to an increase in their volume, and resulting in an increase in foveal thickness [29]. According to other reports [31,32,33], cysts with a lower OCT foveal cyst reflectivity may exhibit degenerative changes that lead to physical fragility and macular edema. These findings suggest that patients with a low OCT reflectivity may experience a short-term improvement in CMT but are also at risk of a worse prognosis for VA or DME recurrence in the long term. Therefore, long-term follow-up studies using OCT reflectivity of foveal cysts as a predictive biomarker for long-term prognosis are needed. Meanwhile, higher OCT reflectivity in the cystic spaces may be related to retinal vessel rupture or endothelial cell death [29]. Since the mechanism leading to higher reflectivity is still unknown and it is unclear as to why the CMT change observed was smaller, more research is needed on this topic; the findings of the present study form a basis for further investigations.

Choi and Kim attempted to find an OCT biomarker associated with anatomical improvement after IVTA in DME unresponsive to bevacizumab [10]; they reported that giant ONL cysts, complete disruption of IS-OS, and >20 HRFs on OCT, as well as worse baseline BCVA and chronic kidney disease (CKD), were related to poor response to IVTA. However, in the present study, none of these parameters were related to the poor response to DEX injection. In the aforementioned study [10], comparisons were made between two groups based on their response to IVTA; poor responders were defined as showing a CMT reduction of <50 μm and a value of >300 μm after IVTA. If these criteria were applied in the present study, all patients except one would be considered good responders, as they showed a CMT reduction >50 μm. In other words, DEX administration exhibited a strong short-term effect, resulting in anatomical improvement after 1 month in most cases, even though all patients were anti-VEGF non-responders in the present study. Compared with their study, differences were noted in the (1) drugs (IVTA vs. DEX), (2) baseline characteristics (patients with CKD were not included in the present study), (3) criteria for steroid responders, and (4) number of OCT scans used for analysis (three vs. five scans), although the same OCT device was used (Spectralis OCT). Therefore, direct comparison of OCT biomarker results between the two studies would be difficult.

Another recent DME study attempted to predict anatomical improvements after DEX injection using OCT parameters [18]; they reported that DRIL affected anatomical improvement after DEX injection. In the present study as well, DRIL was analyzed; however, it was not found to be related to CMT improvement. Most cases in the present study showed improvement in CMT regardless of the presence or severity of DRIL. In their study, (1) the definition of refractory DME was different from that in this study, and (2) naïve and anti-VEGF non-responders were grouped and their data analyzed; thus, the criteria for categorization of participants differed from that of the present study. Therefore, careful interpretation is required. Choi et al. [17] reported that a higher baseline number of HRF was related to poor response; however, HRF was unrelated to outcomes in the present study. In their study, the anti-VEGF and DEX responders were grouped; hence, careful interpretation is required. In addition, many studies have reported conflicting findings on the association between HRF and anatomical response to intravitreal anti-VEGF or steroid injection for DME. The reason for this disparity is attributed to differences in inclusion criteria, definitions of responders and HRF, and differences in OCT models and measurement methods.

There are a few limitations to this study. First, this was a retrospective study with a small sample size. The lack of randomization made it entirely possible that there were baseline CMT differences between the two groups since the two groups were divided based only on the treatment response results. Second, the study period was short because it was designed to find biomarkers predicting short-term CMT changes (1 month) after DEX injection. Third, the definition of responders, drugs administered, OCT type, and analysis methods used in this study may be different from those used in other studies. In particular, the criteria for dividing better responders and lesser responders one month after DEX injection were arbitrary, making it difficult to extrapolate the study results to different settings. Therefore, the results of this study should be interpreted carefully. However, even when the whole group was analyzed without division into two groups, ODN and ODR baseline values showed a significant negative association with CMT change 1 month after DEX injection. Finally, since this study only targeted patients who did not respond to bevacizumab, OCT biomarkers related to other anti-VEGF drugs were not explored. Previous research shows that bevacizumab elicits poorer morphological and functional results compared with other anti-VEGF drugs, such as ranibizumab and aflibercept [34].

Nevertheless, this study is the first to investigate short-term OCT biomarkers by analyzing DRIL, foveal cystoid space, ELM, EZ, HRF, and SRF together to predict the anatomical improvement in patients with DME who received DEX injection due to refractoriness to intravitreal anti-VEGF injection. In addition, the discovery of a new biomarker, i.e., OCT reflectivity of foveal cysts, adds to the significance of this study.

In summary, DEX injection demonstrated short-term effectiveness for the treatment of DME refractory to anti-VEGF therapy, and higher OCT reflectivity in foveal cysts was associated with a lesser decrease in CMT 1 month after DEX injection in IVB non-responders with DME, suggesting the usefulness of this novel OCT biomarker in predicting short-term anatomical responses after DEX injection.

## Figures and Tables

**Table 1:** Baseline characteristics of patients with diabetic macular edema (*n* = 31).

Characteristics	Value
Age (years)	58.77 ± 10.32
Sex (M/F)	13/18
OD/OS	15/16
Previous bevacizumab treatment	4.19 ± 2.07
DR stage	
Proliferative DR Non-proliferative DR	23 (74.2%) 8 (25.8%)
Lens status	
Phakic Pseudophakic	11 (35.5%) 20 (64.5%)
Duration of diabetes mellitus (years)	9.94 ± 9.00
HbA1c (%)	7.47 ± 1.31
BUN (mg/dL)	24.29 ± 16.06
Cr (mg/dL)	1.45 ± 0.95
BCVA (logMAR)	0.46 ± 0.32
IOP (mmHg)	15.42 ± 2.69
CMT (µm)	544.45 ± 131.55

Values are presented as the mean ± standard deviation or as the number (percent). BCVA = best-corrected visual acuity; BUN = blood urea nitrogen; CMT = central macular thickness; Cr = creatinine; DR = diabetic retinopathy; F = female; IOP = intraocular pressure; logMAR = logarithm of the minimum angle of resolution; M = male; OD = oculus dexter; OS = oculus sinister.

**Table 2:** Comparisons of clinical characteristics according to the response to intravitreal dexamethasone treatment.

Characteristics	Better Responder DME (*n* = 22)	Lesser Responder DME (*n* = 9)	*p*-Value
Age (years)	57.73 ± 11.58	61.33 ± 6.06	0.219 ^a^
Sex (M/F)	9/13	4/5	0.583 ^b^
Duration of diabetes mellitus (years)	8.50 ± 7.74	13.44 ± 11.26	0.254 ^a^
HbA1c (%)	7.66 ± 1.46	7.00 ± 0.74	0.356 ^a^
BUN (mg/dL)	26.4 ± 18.07	19.00 ± 8.37	0.569
Cr (mg/dL)	1.57 ± 1.08	1.15 ± 0.43	0.622
BCVA (logMAR)	0.50 ± 0.36	0.36 ± 0.17	0.453 ^a^
IOP (mmHg)	15.05 ± 2.90	16.33 ± 1.94	0.236 ^a^
CMT (µm)	577.68 ± 138.95	463.22 ± 61.27	0.020 ^a^

Values are presented as the mean ± standard deviation or number (percent). BCVA = best-corrected visual acuity; BUN = blood urea nitrogen; CMT = central macular thickness; Cr = creatinine; DME = diabetic macular edema; F = female; IOP = intraocular pressure; logMAR = logarithm of the minimum angle of resolution; M = male. ^a^ Mann–Whitney U test; ^b^ Fisher’s exact test.

**Table 3:** Comparisons of optical coherence tomography parameters in patients with diabetic macular edema according to the response to intravitreal dexamethasone treatment.

Characteristics	Better Responder DME (*n* = 22)	Lesser Responder DME (*n* = 9)	*p*-Value
CMT (µm) Before injection After injection △CMT (µm)	577.68 ± 138.95 287.32 ± 80.20 290.36 ± 123.31	463.22 ± 61.27 365.22 ± 66.27 98.00 ± 40.37	0.004 ^a^ 0.016 ^a^ <0.001 ^a^
DRIL			
Absent Present	2 (9.1) 20 (90.9)	0 (0) 9 (100.0)	1.000 ^b^
Intraretinal cysts in the INL			
Absent Present	1 (4.5) 21 (95.5)	1 (11.1) 8 (88.9)	0.503 ^b^
Intraretinal cysts in the ONL			
Absent Mild Moderate Severe	0 (0) 0 (0) 2 (9.1) 20 (90.9)	0 (0) 1 (11.1) 1 (11.1) 7 (77.8)	0.273 ^b^
Intraretinal cysts in the ONL			
Optical density Optical density ratio	38.54 ± 15.24 1.55 ± 0.88	60.22 ± 21.88 3.46 ± 1.26	0.020 ^a^ <0.001 ^a^
ELM integrity			
Normal Partly disrupted Moderately disrupted Severely disrupted	5 (22.7) 11 (50.0) 4 (18.2) 2 (9.1)	3 (33.3) 4 (44.4) 0 (0) 2 (22.2)	0.422 ^b^
EZ integrity			
Normal Partly disrupted Moderately disrupted Severely disrupted	5 (22.7) 9 (40.9) 4 (18.2) 4 (18.2)	3 (33.3) 4 (44.4) 0 (0) 2 (22.2)	0.575 ^b^
SRF			
Absent Present	13 (59.1) 9 (40.9)	5 (55.6) 4 (44.4)	1.000 ^b^
HRF			
Absent <10 ≥10	0 (0) 10 (45.5) 12 (54.5)	0 (0) 4 (44.4) 5 (55.6)	1.000 ^b^

Values are presented as the mean ± standard deviation or number (percent). CMT = central macular thickness; DME = diabetic macular edema; DRIL = disorganization of the retinal inner layers; ELM = external limiting membrane; EZ = ellipsoid zone; HRF = hyperreflective foci; INL = inner nuclear layer; ONL = outer nuclear layer; SRF = subretinal fluid. ^a^ Mann–Whitney U test; ^b^ Fisher’s exact test.

## Data Availability

The datasets generated during and/or analyzed during the current study are available from the corresponding author on reasonable request.
